# Should flexible ureteroscope be added to our armamentarium to treat stone disease?

**DOI:** 10.4103/0970-1591.44259

**Published:** 2008

**Authors:** Anand Dharaskar, Anil Mandhani

**Affiliations:** Department of Urology, Sanjay Gandhi Post Graduate Institute of Medical Sciences, Lucknow, UP, India

**Keywords:** Cost, flexible ureteroscopy, renal stone, rigid ureteroscopy, ureteric stone

## Abstract

The field of Urology in Medicine has witnessed tremendous advancement in technology and in accordance with it. Endourology has taken a leap ahead in terms of stone management. Most of the stones could be treated with semi-rigid ureteroscopy, percutaneous nephrolithotomy (PNL) and ESWL and some would need Flexible ureteroscopy. Flexible ureteroscopy has been primarily indicated to treat ESWL resistant renal stones but with changes in the technology of incorporating secondary active deflection and availability of laser fibres, its horizon for indications to treat stones is being widened. Though Flexible ureteroscopy is being used to treat stones of various sizes and locations, its cost effectiveness is debatable. Should it be used ubiquitously to treat stones amenable to PNL or ESWL is a big question we need to answer. As of now true indications of Flexible ureteroscopy are limited and there is an urgent need for a randomized trial to compare its efficacy with ESWL and PNL for renal and upper ureteric stones.

## INTRODUCTION

The removal of urinary stones has been greatly facilitated by various endourologic treatment options, including extracorporeal shock wave lithotripsy (SWL), percutaneous nephrolithotomy (PNL), retrograde ureteroscopy, antegrade percutaneous ureteroscopy, and recently, laparoscopic ureterolithotomy.

Advances in instrumentation like availability of semi-rigid and flexible ureteroscopes as well as Holmium: YAG laser have allowed stone fragmentation and removal along the whole course of the urinary tract including the proximal ureter and intrarenal collecting system. Endourology is technology-dependent and with advancing technology comes the peer pressure of having more and more gadgets in one's basket. Ureteroscopy for stones is one of the most commonly performed surgeries by urologists.

Practice of medicine, as also of urology, has faced deviations from the existing norms and guidelines published in peer-reviewed literature. In this era of evidence-based medicine, it is imperative to analyze the existing evidence before instituting treatment to safeguard the interest of the patients. Deviations should not be to keep “in fashion”. Safety of the patients, procedure and the desired surgical outcome in a cost-effective manner should be the foremost concern for a treating surgeon. There has to be enough evidence to regard newer technology superior to the existing one. In this context the present review analyzes the true advantage of having a flexible ureteroscope to treat urinary stones.

## MATERIALS AND METHODS

Articles on ureteroscopy with semi-rigid (SR_URS) and flexible ureteroscopy (F-URS) were searched on Pubmed and reviewed based on the level of evidence. Most of the studies available are of Level 3 and 4 evidence. These studies were analyzed and stone clearance rates were compared based on the location of the stones and the treatment modalities to derive a decision tree to find the use of F-URS in the current practice of urology.

Though proximal ureteric stones and renal stones have been targeted as primary indication for treating stones with F-URS there was no trial either case control or randomized to compare flexible or rigid ureteroscopy for the treatment of such stones.

## HISTORY OF DEVELOPMENT OF URETEROSCOPES

Ureterorenoscopy was first done in 1912 when Dr. Young inadvertently entered the ureter of a male patient with posterior urethral valves.[1] There was not much progression until 1959 when Professor Harold H. Hopkins introduced the rod-lens optical system and fiberoptic cold-light source was developed by Karl Storz.[2]

The rod lens system of endoscopy was next used by Dr. Goodman in 1977 when he used 11Fr pediatric cystoscope to examine the distal ureter in women.[3] Lyon *et al*. used a pediatric ureteroscope to treat ureteral urothelial carcinoma in a female in 1978.[4] This led to the first true ureteroscope especially designed for the ureter and kidney, which was introduced in 1980.[5] Despite improvements in the quality of radiological imaging, development of molecular markers of malignancy, extracorporeal and percutaneous treatments of upper tract pathology, ureteroscopy still is an important tool to reckon with.

## RIGID VERSUS FLEXIBLE URETEROSCOPY

While rigid ureteroscopes are not used anymore owing to availability of better and versatile semi-rigid ureteroscopes, flexible ureteroscopy is making inroads in the practice of urology. In endourology, success of the procedure is attributed to a wide array of instruments but it is not always possible to have all the gadgets in one's armamentarium. When the cost of the treatment is a major constraint then we should think sensibly to deliver healthcare with efficacy and the safety based on the evidence available.

The advantages of the flexible ureteroscopes are their ability to safely negotiate the angulations of the ureter, and they can access the entire upper collecting system in over 90% of patients with active and secondary passive deflection, and now with secondary active deflection.[6–8] Rigid and flexible ureteroscopes have been used in a complementary fashion to access the entire upper urinary tract.

Though the standard approach of distal two-thirds ureteric stones is SR-URS, controversy continues for proximal ureteric stones as to whether they are best treated with F-URS or SR-URS, particularly in reference to cost of the procedure. Common perceived view is that F-URS is a very good way to treat smaller renal stones too but in actual sense this may not be true. We would discuss these two aspects in detail to draw some conclusions based on the best evidence available.

## TREATING PROXIMAL URETERAL STONES: SEMI-RIGID OR FLEXIBLE URETEROSCOPY

Although American Urological Association (AUA) recommendations favor F-URS for upper ureteric stones of more than 1 cm and ESWL for stones less than 1 cm, there is emerging evidence that upper ureteric stones can safely be dealt with semi-rigid ureteroscope too.^9^ There are very few case series on F-URS. Sofer and colleagues reported stone-free rates for proximal ureteric stones (mean stone size of 11.3 mm) of 97% using both SR-URS and F-URS with 0.35% for major complications (ureteral strictures).[10] Bagely DH reported 95% success with F-URS for stones less than 1 cm for proximal ureteric stones.[11]

Though access to the upper ureter with a semi-rigid ureteroscope sometimes could be challenging but with certain maneuver and expertise, upper ureter can be accessed safely and efficiently with a 7.5F SR-URS in nearly all patients.[12–16] Lower abdominal pressure can be helpful to negotiate passage of the endoscope over the iliac vessels or to place the laser fiber on stones using flexible ureteroscope with holmium.[12]

Though there is no head to head comparison between F-URS and SR-URS for any size of the upper ureteric stones, there is one randomized trial between ESWL and SR-URS for large proximal ureteric stones. Accessibility of the semi-rigid ureterorenoscope for impacted upper ureteral stones 1.51±0.05 cm was 95.1% and the stone-free rate achieved after one sitting was 92%.[15] The initial stone-free rate in the SR-URS group was better than that of the SWL group (*P* _ 0.003). The average cost in the SR-URS group appeared to be lower than in the SWL group. This study demonstrated that SR-URS achieved excellent results for upper ureteral calculi greater than 1 cm.

Cheung *et al*. reported that SR-URS with Holmium: YAG laser lithotripsy was safe and effective for treating calculi larger than 10 mm in longest diameter at all levels in an outpatient setting with success rate of 93%.[16] These rates are equal to the stone-free rates with F-URS which was only done for less than 1 cm and 11 mm (mean size) stones[10,11] [Table 1].

**Table 1:** Comparison of results and complication rate between F-URS and SR- URS

Author	Ureteroscope	Stone-free rate (%)	Complication (%)
Tawfiek, 1999 (13)	SR-URS	98%	3
Soichi, 2000 (14)	SR-URS	96.2%	0
Sofer, 2002 (10)	F-URS	97%	-
Bagely, 1990 (11)	F-URS	92%	0 (major)

Thus, SR-URS should be considered as first-line therapy for large proximal ureteral stones.[15] There is another option available of antegrade removal for large impacted proximal ureteric stones with the success rates ranging from 98.5-100% with no intra and postoperative complications.[17,18

## DISTAL URETERIC STONES AND URETEROSCOPY

Ureteroscopy for the distal ureter has become a routine procedure for most urologists. Success rates can approach 100% for fragmentation and stone removal in the distal ureter, technical complications from instrumentation and use of energy sources are avoidable, and complication rates approach 0%.[19,20] A randomized trial and review of published series from 1990 to 2001 on the treatment of distal ureteral stones revealed success rates of 94.9% (range 90.4-100%) with ureteroscopy versus 87.2% (range 85.6-89.2%) after ESWL.[19,21] Ureteroscopy was found to be significantly better in terms of operative time, fluoroscopy time, and time to achieve stone-free status.

There is no trial published comparing F-URS with SR-URS for the lower ureteric stones too. Flexible URS is more difficult to use in the lower ureter because of its tendency to buckle into the bladder.[11] Therefore with the present evidence available in the literature, SR-URS scores over F-URS on ease of the procedure and the cost for treating stones in the lower two-thirds of the ureter.

## FLEXIBLE URS IN TREATING RENAL CALCULI

Extracorporeal shock wave lithotripsy is the mainstay of treatment for calculi 20 mm or less in size. Higher re-treatment rates after SWL for stones > 20 mm have led the AUA panel to recommend the more invasive percutaneous approach for such stones.[9] The majority of urinary calculi between 10 and 20 mm are still managed initially with SWL.[9] As F-URS cannot reach the pelvicalyceal system, there is an increasing trend towards using F-URS for renal stones over SWL. There is only one randomized trial comparing F-URS with SWL for stones less than 1 cm. This study failed to demonstrate a statistically significant difference in stone-free rates between SWL and F-URS for the treatment of small lower pole renal calculi. However, SWL was associated with greater patient acceptance and shorter convalescence.[22] There are reports of treating larger stones more than 2 cm in size in the kidney with flexible ureteroscopy but the success rate was 77% at first sitting and 91% on second look ureteroscopy.[23,24] Treating large calculi required second look URS in 53% and third look URS in 33% of cases adding to the tremendous cost and discomfort to the patients.[23] In another prospective study success rate using F-URS for renal stone size of 1-2 cm was 80% and only 50% for stones greater than 2 cm.[24]

These stone-free rates for stones more than 2 cm are far less than those reported with PNL. As of now there is no evidence which suggests that F-URS should be used primarily for renal stones though it may be a good alternative for SWL-resistant upper tract stones less than 2 cm in size.

## COMPLICATIONS OF URETEROSCOPY

Miniaturization of the instruments, better optics and visualization and Holmium laser lithotripsy have a significant impact on the complications of URS. The universally accepted major complications rate of less than 1% is for both F-URS and SR-URS. In one of the largest ever experiences from India the rate of total morbid complications e.g. ureteric avulsion, severe urosepsis necessitating ventilatory support and hemodialysis in more than 12000 patients was .01%.[25]

## DURABILITY OF FLEXIBLE URETEROSCOPES

Smaller flexible ureteroscopes have traditionally been more fragile than their larger counterparts, posing cost challenges for the hospitals. The currently available flexible ureteroscopes less than 9Fr are fragile instruments. The average of number of cases done with F-URS before repair ranged from 3.25 for the Wolf™ 7325 to 14.4 for the ACMI™ DUR™-8 Elite.[26] There is great variability among makes and models in the marketplace. Until durability improves the use of these endoscopes, F-URS may well be at institutions with the resources necessary to purchase several and keep them operational. In another study F–URS was used for an average of 3 to 13 h before it required repair. The most fragile part of these instruments was the deflection unit.[26]

## COST COMPARISON BETWEEN F-URS AND SR-URS

Healthcare cost has become an important issue to determine management strategy. In one interesting study the cost of F-URS instrument based on initial purchase and maintenance cost was 1000 US $ per case if flexible ureteroscope lasted for 25 cases.[27] This excludes all other expenditure to carry out the procedure. After the initial purchase the cumulative cost of the disposables overtakes the cumulative cost of purchase and upkeep of the ureteroscope.[28] Though there is no head to head comparison, this is far too high than the cost per case done with SR-URS. Similarly, the cost of ESWL factoring the need of retreatments, ancillary procedures, office visits due to subsequent pain, and re-admission to emergency rooms, was significantly higher than ureteroscopy done with SR-URS.[29]

## FUTURE RESEARCH

**Figure 1:**
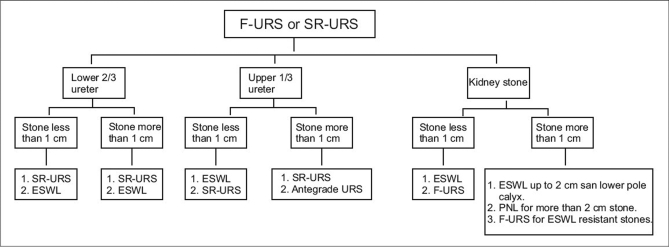
Flowchart depicting role of flexible ureteroscopy in treating stone disease

We should endeavor in future to find answers to the debatable questions by conducting randomized trial for upper ureteric stone and renal stones more than 1 cm in size treated with either F-URS or SR-URS and F-URS and PNL respectively. Till that happens adding this instrument in the armamentarium would not be a cost-effective proposition to treat stones [Figure 1]. Patients who actually need F-URS should be referred to the larger centers where this facility is available. At present the only situations where F-URS scores over SR-URS are ESWL refractory renal stones and diagnostic evaluation of upper tracts.
